# Prevalence and Associated Factors of Overweight or Obesity and Abdominal Obesity in Iranian Population: A Population-based Study of Northwestern Iran

**Published:** 2018-10

**Authors:** Jafar Sadegh TABRIZI, Homayoun SADEGHI-BAZARGANI, Mostafa FARAHBAKHSH, Leila NIKNIAZ, Zeinab NIKNIAZ

**Affiliations:** 1. Tabriz Health Services Management Research Center, Faculty of Management and Medical Informatics, Tabriz University of Medical Sciences, Tabriz, Iran; 2. Dept. of Statistics and Epidemiology, Faculty of Health, Tabriz University of Medical Sciences, Tabriz, Iran; 3. Research Center of Psychiatry and Behavioral Sciences, Tabriz University of Medical Sciences, Tabriz, Iran; 4. Tabriz Health Services Management Research Center, Tabriz University of Medical Sciences, Tabriz, Iran; 5. Liver and Gastrointestinal Disease Research Center, Tabriz University of Medical Sciences, Tabriz, Iran

**Keywords:** Adults, Overweight, Obesity, Abdominal obesity

## Abstract

**Background:**

This population-based study aimed at investigating the prevalence and associated factors of over-weight /obesity and abdominal obesity in Iran.

**Methods:**

The study population consists of 2818 inhabitant of the urban and regional area of East-Azerbaijan, Iran in 2015. The weight, height and waist circumferences were measured and the body mass index (BMI) and conicity index were calculated. The ANCOVA and logistic regression were used for statistical analysis.

**Results:**

Prevalence of overweight, obesity and abdominal obesity was 39.6%, 24%, and 76.4% respectively. Women showed the higher prevalence of obesity (32.2%) and abdominal obesity (81.4) than men (obesity: 15.1%; abdominal obesity: 68.6%). Age, marriage and family history of obesity were independent predictors of obesity in the population (*P*< 0.001). In men and women, nonsmokers (*P*<0.01) and subjects having more than two kids (*P*< 0.001) were also more expected to be overweight or obese and abdominally obese respectively.

**Conclusion:**

More actions mostly focusing on education and physical activity levels, and changing eating habits are required.

## Introduction

Obesity is one of the major community health problems and its occurrence is notably growing in developed and developing countries (1, 2). Based on recent reports, the rate of obesity has tripled during the last two decades in developing countries (3). Iran has been no exception. According to the latest report of WHO, more than half of the Iranian adults are overweight or obese (4).

In adults, the most prevalent and practical indicator for evaluation of overweight and obesity is Body Mass Index (BMI) (5). Obesity and abdominal obesity are the main risk factors for developing cardiovascular diseases (CVD), diabetes, hypertension and dyslipidemia (6–9).

Increasing of obesity prevalence is related to the significant modification in the lifestyle in particular dietary habits and limited physical activity in both urban and rural regions (10, 11). Some of the ethnic dimensions underlying causes of obesity are low socioeconomic status, genetic factors, and the different eating patterns (12–15). In Iran, family history of obesity, aging, early marriage, low physical activity, parity, and education are responsible for both obesity and central obesity in the urban population (16).

There is no recent data available for prevalence and associated factors of overweight or obesity and abdominal obesity in Iran. This population-based investigation focused mainly on providing the estimates of obesity in Iran and also evaluating its association with various risk factors in the Iranian adult population in the urban and regional areas.

## Materials and Methods

The data for this study were collected in 2015 as a part of the major Lifestyle Promotion Project (LPP) (17, 18) conducted in the districts of East Azerbaijan (urban and regional parts), one of the largest provinces of Iran. This study performed by probability proportional to size (PPS) multistage stratified cluster sampling through which 150 clusters selected. In each cluster, 20 participants (15–65 yr) were enrolled (3000 participants). All procedures performed in this study were in line with the ethical values of the Ethics Committee of Tabriz University of Medical Science and Informed consent was obtained from all participants included in the study.

Exclusion of incomplete questionnaire yielded 2818 final sample, subjected to statistical analysis. The final sample consists of 1370 and 1448 residents in the capital city (Tabriz) regional areas (including Marand, Mianeh, Varzegan, Khodafarin, Bonab, Osku, and Ilkhichi) respectively.

International Physical Activity Questionnaire (IPAQ) which the validity of the translated form was evaluated in the previous study on Iranian subjects (19). Dietary assessment is performed by means of quantitative food frequency questionnaires (20).

BMI was calculated from height and weight data as kg/m^2^. Overweight was defined as BMI 25–29.9 kg/m^2^ and obesity as BMI≥30 kg/m^2^. Waist circumferences (cm) were measured in duplicate with an anthropometric tape at the minimum circumference between the iliac crest and the rib cage and conicity index was calculated as follow: Conicity index = waist circumference in meters/(0.109*square root of weight in kg/height in meters).

### Statistical analysis

SPSS ver.18 (Chicago, IL, USA) Statistical computer software was used for all statistical analyses. Means and standard deviations (SD) were calculated for continuous variables, and proportions were calculated for categorical variables. Between-group comparisons were made by independent *t-*test, chi-square test, and ANCOVA. The multinomial logistic regression models were used to examine the relationships between over-weight/obesity/abdominal obesity and associated factors by adjusting for covariates. All analyses were performed for men and women separately since the literature usually reports gender differences for factors associated with obesity. A significance level of 0.05 was used.

## Results

The prevalence of overweight, obesity and central obesity in the urban and regional areas is shown in Table 1. The rates of age, marital, occupational, educational and physical activity status, smoking habit, overweight, obesity and abdominal obesity were significantly different in the urban and regional areas (*P*<0.05).

**Table 1:** The prevalence of overweight, obesity and abdominal obesity in urban and regional areas

	***Urban (n= 1370)***	***Regional(n= 1448)***	***Total (n= 2818)***
Age, % (n)^*^			
15–25	5.1 (71)	8.5 (123)	6.8 (191)
26–35	18.7 (257)	22.2(322)	20.5(578)
36–45	32 (439)	28.4(412)	30.2(851)
46–55	24 (330)	23.2(335)	23.8(671)
56–65	19.8 (272)	17.7(256)	18.7 (527)
**Marital status, % (n)**			
Married^*^	89.1 (1220)	85.5 (1239)	87.1 (2454)
Occupational status, % (n)^*^			
Employed or self employed	39.2 (537)	42.4 (614)	40.9 (1153)
Student	5.6 (77)	6.7 (97)	6.2 (175)
Unemployed	55.2 (756)	50.9 (737)	52.9 (1490)
Educational status, % (n)^*^			
Illiterate	11.2 (153)	14.8 (214)	13.0 (366)
Under graduate	67.1 (920)	71.2 (1031)	69.2 (1950)
College	21.7 (279)	14.1 (204)	17.8 (501)
Smoking habit, % (n)^*^			
yes	9.5 (130)	12.7 (184)	11.1 (313)
Occasionally	1.3 (18)	2.0 (28)	1.7 (48)
No	89.1 (1221)	85.4 (1236)	87.2 (2457)
Physical activity, % (n)^*^			
Inactive	43.3 (593)	18.1 (262)	30.3 (854)
Minimally active	34.8 (477)	29.2 (423)	31.9 (899)
Health enhancing activity	21.9 (300)	52.7 (763)	37.7 (1062)
Prevalence of overweight, % (n)^*^	41.6 (570)	37.7 (546)	39.6 (1116)
Prevalence of obesity, % (n)^*^	25.7 (352)	22.4 324)	24.0 (676)
Prevalence of abdominal obesity, % (n)^*^	76.3 (1045)	74.1 (1074)	75.2 (2119)

* (*P*<0.05), differences tested by chi-square test

The prevalence of overweight, obesity and central obesity was significantly higher in the urban areas (*P*<0.01); however, health-enhancing physical activity and smoking were significantly more prevalent in the regional areas (*P*<0.001).

Table 2 illustrates the prevalence of overweight, obesity, and abdominal obesity by age and sex. The prevalence of overweight, obesity and abdominal obesity were higher in middle**-**aged adults compared to young adults. Moreover, the prevalence of central obesity in normal weight subjects was 15.3% (men: 16.7%, women: 14.1%).

**Table 2:** The prevalence of overweight, obesity, and abdominal obesity by age and sex

***Variables***	***Men (n=1368)***	***Women (n=1450)***	P-value^*^
Weight, kg, (mean±SD)^†^	76.4±13	69.7±13	<0.001
15–25	64.9±12.7	59±12.1	0.005
26–35	74.7±13	64.3±12.4	<0.001
36–45	80.1±12.4	70.6±12.5	<0.001
46–55	78.8±11.9	72.8±12.6	<0.001
56–65	77.1±11.8	73.1±12	<0.001
*P*-value	<0.001	<0.001	
Height, cm, (mean±SD)^†^	171.9±8.4	157.2±13.7	<0.001
15–25	171.9±9	160.2±7.5	<0.001
26–35	174.8±7.4	159.4±14.6	<0.001
36–45	173.6±8.4	157.5±14.8	<0.001
46–55	171.4±9	157.3±16.2	<0.001
56–65	169±7.5	153.6±18.7	<0.001
*P*-value	<0.001	<0.001	
BMI, kg/m^2^, (mean±SD)^†^	25.9±4.7	28±5.6	<0.001
15–25	22.2±4.2	22.9±4.5	0.127
26–35	24.5±4	25±4.4	0.09
36–45	26.7±5	28±5.3	<0.001
46–55	27±4.6	29.4±4.9	<0.001
56–65	27±4.1	30.4±6.2	<0.001
*P*-value	<0.001	<0.001	
Waist circumference, cm, (mean±SD)^†^	91.6±12.3	91.2±12.7	0.44
15–25	79.1±10.2	77.7±12.9	0.42
26–35	86.3±11.4	84.4±11.8	0.06
36–45	92.7±12.4	90.7±11.1	0.01
46–55	94.9±10.5	94.2±12.1	0.49
56–65	96.9±10.8	98.5±10.4	0.027
*P*-value	<0.001	<0.001	
Prevalence of overweight, %^‡^	42.2	37.1	0.01
15–25	16.1	16.4	0.56
26–35	30.8	28.6	0.62
36–45	44.6	42.3	0.55
46–55	49.4	40.7	0.046
56–65	52.3	37.5	0.001
*P*-value	<0.001	<0.001	
Prevalence of obesity, %^‡^	15.1	32.2	<0.001
15–25	2.7	7.2	0.16
26–35	8.6	15.1	0.039
36–45	18.9	29.5	0.001
46–55	18.2	42.6	<0.001
56–65	19	47.5	<0.001
*P*-value	<0.001	<0.001	
Prevalence of abdominal obesity, %^‡^	68.6	81.4	<0.001
15–25	20.0	36.2	<0.04
26–35	43.9	63.9	<0.001
36–45	75.3	84.0	<0.001
46–55	81	90.7	<0.001
56–65	86.6	96.6	<0.001
*P*-value	<0.001	<0.001	

† Differences tested by unpaired Student’s *t*-test//

‡ Differences tested by chi-square test

Odds ratios for overweight/obesity and abdominal obesity for demographic, socioeconomic and lifestyle factors were presented in Table 3. Results of logistic regression analysis, which controlled to confounders (age, marriage, residency area, employment, education, smoking and physical activity status, the number of kids and the family history of obesity) showed that in both sexes, higher age was the risk of over-weight/obesity and abdominal obesity. In married men, the risk of being overweight/obese and centrally obese was more than twice as high compared to single ones. Moreover, nonsmokers and subjects having the family history of obesity were also more expected to be overweight or obese and centrally obese than the others. In addition, 15% and 58% higher risk of obesity and abdominal obesity was seen in women with health-enhancing physical activity.

**Table 3:** Logistic regression analysis for the association of overweight/obesity/abdominal obesity and demographic, socio-economic, lifestyle factors

	**Men(n=1368)**		**Women(n=1450)**	
	***Overweight/ Obesity Adjusted OR (95% CI)***	***Abdominal obesity Adjusted OR (95% CI)***	***Overweight/ Obesity Adjusted OR (95% CI)***	***Abdominal obesity Adjusted OR (95% CI)***
Age groups				
15–25	1.00	1.00	1.00	1.00
26–35	1.85 (0.83,4.14)	1.93 (0.88, 4.20)	2.09 (0.98, 4.45)	2.15 (1.08, 4.27)^*^
36–45	4.24 (1.77, 10.14) ^***^	6.14 (2.59, 14.53) ^***^	6.43 (3.02,13.70) ^***^	6.18 (3.06, 12.49) ^***^
46–55	4.6 (1.90,11.27) ^***^	9.53 (3.88, 23.39) ^***^	12.5 (5.71, 27.42) ^***^	11.03 (5.14, 23.69) ^***^
56–65	6.24 (2.49, 15.63) ^***^	11.31 (4.37, 29.25) ^***^	15.3 (6.61,35.41) ^***^	44.95 (14.33, 141.04) ^***^
Residential place				
Urban	1.00	1.00	1.00	1.00
Rural	0.98 (0.74, 1.29)	1.24 (0.88, 1.74)	0.82 (0.63, 1.07)	0.95 (0.74, 1.22)
Marital status				
Single	1.00	1.00	1.00	1.00
Married	2.24 (1.35, 3.73) ^***^	2.09 (1.24, 3.15) ^***^	1.76 (1.03, 3.01) ^*^	1.72 (1.10, 3.04) ^*^
Occupational status				
Employed	1.00	1.00	1.00	1.00
Student	1.10 (0.49, 2.44)	1.15 (0.29, 4.63)	0.61 (0.21, 1.78)	2.07 (0.70, 6.08)
Unemployed	1.93 (1.03, 2.99) ^**^	0.81 (0.43, 1.52)	1.13 (0.68, 1.87)	0.87 (0.53, 1.41)
Educational status				
Illiterate	1.00	1.00	1.00	1.00
Under graduate	1.40 (0.76, 258)	1.50 (0.78, 2.89)	1.55 (0.9, 1.99)	0.94 (0.62, 1.43)
College	1.42 (0.73, 2.77)	1.35 (0.64, 2.82)	0.99 (0.57, 1.74)	1.41 (0.83,2.41)
Smoking habit				
yes	1.00	1.00	1.00	1.00
Occasionally	1.64 (0.72, 3.76)	0.59 (0.23, 1.54)	2.30 (0.20,26.00)	1.09 (0.06, 19.23)
No	1.64 (1.19, 2.28) ^**^	1.69 (1.17, 2.44) ^**^	2.57 (0.54, 12.13)	3.68 (0.61, 22.01)
Physical activity				
Inactive	1.00	1.00	1.00	1.00
Minimally active	1.00 (0.68, 1.46)	1.21 (0.80, 1.83)	0.90 (0.65, 1.24)	1.06 (0.73, 1.55)
Health enhancing activity	1.06 (0.72, 1.55)	1.32 (0.86, 2.01)	0.88 (0.63, 1.23)	1.58 (1.06, 2.30) ^*^
Family history of obesity				
No	1.00	1.00	1.00	1.00
Yes	1.93 (1.13, 3.31) ^*^	1.90 (1.34, 2.69) ^***^	1.78 (1.30, 2.43) ^***^	2.03 (1.22, 3.38) ^***^
Number of kids				
0–1	1.00	1.00	1.00	1.00
≥2	0.65 (0.03, 11.6)	1.07 (0.66, 1.72)	1.59 (1.23, 2.05) ^***^	2.08 (1.35, 3.19) ^***^

* *P* < 0.05

** *P* < 0.01

*** *P* < 0.001

Multiple logistic regressions considering the simultaneous effect of all the explanatory variables

The energy and macronutrient intake of men and women in different subgroups of anthropometric measures are represented in Table 4. In both sexes, after adjusting for age, educational and professional profile, overweight/obese men and women presented a lower energy intake from fat than their leaner counterparts. Additionally, men with central obesity were existing higher and lower energy intake from carbohydrates and proteins respectively.

**Table 4:** Energy and macronutrient intake of men and women in different subgroups of anthropometric measures

	***BMI***			***Abdominal obesity***		
	***BMI<25***	***BMI≥25***	P-value^*^	***CI<0.5***	***CI≥0.5***	P-value^*^
Men						
Energy intake (Kcal/day)	3695±956	3468±942	0.18	3719±966	3558±960	0.11
Energy intake from carbohydrate (%)	48.6±8.3	50.5±8.5	0.14	51.6±5.2	55.1±7.9	0.03
Energy intake from protein (%)	19.2±3.4	19.5±3.0	0.40	20.3±2.2	19.3±3.3	0.04
Energy intake from fat (%)	32.2±7.1	30±7.0	0.04	28.4±5.4	25.6±7.3	0.11
Women						
Energy intake (Kcal/day)	2941±755	3115±825	0.28	3090±768	3020±723	0.60
Energy intake from carbohydrate (%)	49.4±8.0	51.8±9.4	0.12	49.3±8.4	48.4±7.9	0.41
Energy intake from protein (%)	18.9±3.0	19.1±3.2	0.60	19.1±3.0	18.7±3.4	0.45
Energy intake from fat (%)	31.7±7.3	29.1±8.3	0.048	31.6±8.5	32.9±6.7	0.15

BMI: Body Mass Index; CI: Conicity Index //

* ANCOVA, adjusted for age, employment status, marital status, education, residential place, smoking habit, physical activity and number of kids

## Discussion

We provided population-based estimates of obesity and associated factors in East Azerbaijan, Iran. Around 63.6% of the participants were either overweight or obese. This rate reflects a significant national obesity problem. In this study, higher rates of overweight and obesity were observed when compared with estimates for the Spanish (17%) (21) and Turkish (19%) (22) adult population. The results of current study are comparable to those reported by the National Health and Nutrition Examination Survey [NHANES] (more than one-third of adult were obese)(23), and in Pakistan (25.0%)(24).

According to the results of current study, 75.2% (81.4% in women and 68.6% in men) of the population had of central obesity. Abdominal obesity is a health problem in Iran (25–27). The prevalence of abdominal obesity reported being 39.2% in Rio de Janeiro(28), 24.1% in Egypt(29), 30.5% in Australia (30), and 64.4% in Oman (31).

Additionally, in agreement with other studies, overweight/obesity was significantly more prevalent in the urban than regional areas with rates of 67.3% versus 60.1%(32; 33). Compared to the previous report in Iran (34) and based on the results of this study, the prevalence of over-weight and obesity is also rapidly increasing among regional residents along with urban ones which have different resons (35–37).

The prevalence of overweight/obesity and central obesity was even more alarming (76.4%) especially among females (69.3% and 83.2%). These results were dissimilar to the studies in China (38) and Japan (39); however, it was in accordance with other provincial and national studies(1, 16, 40) and consistent with the findings from studies performed in some other countries (16, 40, 41). The number of pregnancies, the amount of weight gain in each pregnancy (42), physical activity pattern and more importantly dietary habits, the social norms and gender responsibilities in traditional communities, where females are perceived primarily as child bearers, may contribute to this gender difference.

The results of this study showed that obesity is increasing sharply with age in both sexes which is consistent with the data from other countries (43–46). In this study, the alarming point is that 45% of women in the 46–65 yr old age group were obese. Weight gain after menopause, age-related lower metabolic consumption (47), the decrease in the level of physical activity with age(48) and decrease in height as a person ages(33) were probably the relative causes of increasing obesity with increasing age. In addition, in consistence with other published studies, age has been considered as a predictor of abdominal obesity (49–52).

Our data indicated that being single was linked with the lower frequency of overweight/obesity and abdominal obesity in both sexes, confirming results of other studies (1, 53).

In this study, a high risk of overweight/obesity among unemployed men was also recognized. Unemployed subjects may have lower opportunities for physical activity and, on the other side, may have irregular eating patterns (54). Unlike other studies, there was no connection between education and obesity in this study (49, 55–58).

Among men, nonsmokers were also more likely to be overweight or obese and abdominally obese than the others. The smoking-BMI connection may be due to the effect of smoking on physiological processes that cause the changes in appetite, food choices, and basal metabolic rates (BMR) (59).

Despite our results, low physical activity and central obesity were associated in other studies reported from Iran and other countries (58; 60). This observation may be due to this fact that in recent years, many interventions to improve physical activity have been carried out in women between 18 and 65 yr of age in Iran and it seems that obese women tended to increase their physical activity in response to these intervention programs (61).

The connection between family size and the number of children and the prevalence of obesity have been reported in previous studies (32, 45, 62). This may be attributed to the age, pregnancy and breastfeeding, the situations most women suppose that it is healthier both for mother and baby to raise their energy intake (63).

Subjects with overweight/obesity and centrally obese subjects reported higher energy intake from fat and carbohydrate. Moreover, abdominally obese men reported lower protein intake than the others. The result of one study showed lower energy intake from proteins in abdominally obese men. High protein diet may increase the thermogenic response and decrease the calorie intake by satiety (64, 65). On the other hand, a high-carbohydrate diet causes hyperinsulinemia, high serum triglycerides and low HDL-C levels (66) which may cause central obesity. A low-fat, high-carbohydrate diet mainly consisting of highly refined grains and products with added sugar which may increase prevalence of obesity (67).

## Conclusion

There is high prevalence of overweight/obesity (63.6%) and abdominal obesity (75.2%) among adults. More longitudinal study is desired to investigate the association between socio-demographic factors and obesity.

## Ethical considerations

Ethical issues (Including plagiarism, informed consent, misconduct, data fabrication and/or falsification, double publication and/or submission, redundancy, etc.) have been completely observed by the authors.

## References

[1] Janghorbani M Amini M Willett WC (2007). First nationwide survey of prevalence of overweight, underweight, and abdominal obesity in Iranian adults. Obesity (Silver Spring), 15: 2797–2808. 18070771 10.1038/oby.2007.332

[2] Ayatollahi S Ghoreshizadeh Z (2010). Prevalence of obesity and overweight among adults in Iran. Obes Rev, 11: 335–337. 20202133 10.1111/j.1467-789X.2010.00725.x

[3] Hossain P Kawar B El Nahas M (2007). Obesity and diabetes in the developing world—a growing challenge. N Engl J Med, 356: 213–215. 17229948 10.1056/NEJMp068177

[4] Alwan A (2011). Global status report on noncommunicable diseases 2010: World Health Organization.

[5] Gill T (2006). Epidemiology and health impact of obesity: an Asia Pacific perspective. Asia Pac J Clin Nutr, 15 Suppl:3–14. 16928656

[6] Molarius A Seidell J (1998). Selection of anthropometric indicators for classification of abdominal fatness Ð a critical review. Int J Obes Relat Metab Disord, 22: 719–727. 9725630 10.1038/sj.ijo.0800660

[7] Kragelund C Omland T (2005). A farewell to body-mass index? Lancet, 366: 1589–1591. 16271629 10.1016/S0140-6736(05)67642-8

[8] Sowers JR (2003). Obesity as a cardiovascular risk factor. Am J Med, 115 Suppl 8A:37S–41S. 14678864 10.1016/j.amjmed.2003.08.012

[9] Tabrizi JS Sadeghi-Bazargani H Farahbakhsh M (2016). Prevalence and Associated Factors of Prehypertension and Hypertension in Iranian Population: The Lifestyle Promotion Project (LPP). PLoS One, 11: e0165264. 27783691 10.1371/journal.pone.0165264 PMC5082665

[10] Marinou K Tousoulis D Antonopoulos AS (2010). Obesity and cardiovascular disease: from pathophysiology to risk stratification. Int J Cardiol, 138: 3–8. 19398137 10.1016/j.ijcard.2009.03.135

[11] Tee E (2002). Obesity in Asia: prevalence and issues in assessment methodologies. Asia Pac J Clin Nutr, 11: S694–S701.

[12] Bazzaz JT Shojapoor M Nazem H (2010). Methylenetetrahydrofolate reductase gene polymorphism in diabetes and obesity. Mol Biol Rep, 37: 105–109. 19437140 10.1007/s11033-009-9545-z

[13] Hasani-Ranjbar S Amoli MM Tabatabaei-Malazy O (2012). Effect of adiponectin gene polymorphisms on waist circumference in patients with diabetes. J Diabetes Metab Disord, 11: 14. 23497697 10.1186/2251-6581-11-14 PMC3598167

[14] Lal A Moodie M Ashton T (2012). Health care and lost productivity costs of overweight and obesity in New Zealand. Aust N Z J Public Health, 36: 550–556. 23216496 10.1111/j.1753-6405.2012.00931.x

[15] Tabatabaei-Malazy O Hasani-Ranjbar S Amoli MM (2010). Gender-specific differences in the association of adiponectin gene polymorphisms with body mass index. Rev Diabet Stud, 7: 241–246. 21409316 10.1900/RDS.2010.7.241 PMC3061614

[16] Hajian-Tilaki K Heidari B (2007). Prevalence of obesity, central obesity and the associated factors in urban population aged 20–70 years, in the north of Iran: a population-based study and regression approach. Obes Rev, 8: 3–10. 17212790 10.1111/j.1467-789X.2006.00235.x

[17] Tabrizi JS Farahbakhsh M Sadeghi-Bazargani H (2016). Introducing the Objectives, Procedures and Structure of Lifestyle Promotion Project (LPP): Phase I. Depiction of Health, 7: 1–7.

[18] Tabrizi JS Farahbakhsh M Sadeghi-Bazargani H (2018). Prevention and Control of Non-communicable Diseases in Iranian Population: Life Style Promotion Project Phase II: Study Protocol. Iran J Public Health, 47:1397–1405. 30320015 PMC6174058

[19] Vasheghani-Farahani A Tahmasbi M Asheri H (2011). The Persian, last 7-day, long form of the International Physical Activity Questionnaire: translation and validation study. Asian J Sports Med, 2:106–116. 22375226 10.5812/asjsm.34781 PMC3289200

[20] Nikniaz L Tabrizi JS Sadeghi-Bazargani H (2017). Reliability and relative validity of short-food frequency questionnaire. Br Food J, 119:1337–1348.

[21] Coll JL Bibiloni M Salas R (2015). Prevalence and related risk factors of overweight and obesity among the adult population in the Balearic Islands, a Mediterranean Region. Obes Facts, 8: 220–233. 26159577 10.1159/000435826 PMC5644906

[22] Jafari-Adli S Jouyandeh Z Qorbani M (2014). Prevalence of obesity and overweight in adults and children in Iran; a systematic review. J Diabetes Metab Disord, 13: 121. 25610814 10.1186/s40200-014-0121-2 PMC4301060

[23] Ogden CL Carroll MD Kit BK (2012). Prevalence of obesity in the United States, 2009–2010. NCHS Data Brief, (82):1–8. 22617494

[24] Jafar TH Chaturvedi N Pappas G (2006). Prevalence of overweight and obesity and their association with hypertension and diabetes mellitus in an Indo-Asian population. CMAJ, 175: 1071–1077. 17060656 10.1503/cmaj.060464 PMC1609152

[25] Amani R (2007). Comparison between bioelectrical impedance analysis and body mass index methods in determination of obesity prevalence in Ahvazi women. Eur J Clin Nutr, 61: 478–482. 17063145 10.1038/sj.ejcn.1602545

[26] Azadbakht L Mirmiran P Shiva N Azizi F (2005). General obesity and central adiposity in a representative sample of Tehranian adults: prevalence and determinants. Int J Vitam Nutr Res, 75: 297–304. 16229347 10.1024/0300-9831.75.4.297

[27] Veghari G (2004). Obesity among mothers in rural golestan. J Sch Public Health Inst Public Health Res, 3: 21–28.

[28] Ramos de Marins VM Varnier Almeida RM Pereira RA Barros MB (2001). Factors associated with overweight and central body fat in the city of Rio de Janeiro: results of a two-stage random sampling survey. Public Health, 115: 236–242. 11429722 10.1038/sj/ph/1900763

[29] Lfotouh A Soliman L Mansour E (2008). Central obesity among adults in Egypt: prevalence and associated morbidity. East Mediterr Health J, 14:57–68. 18557452

[30] Dalton M Cameron A Zimmet P (2003). Waist circumference, waist–hip ratio and body mass index and their correlation with cardiovascular disease risk factors in Australian adults. J Intern Med, 254: 555–563. 14641796 10.1111/j.1365-2796.2003.01229.x

[31] Al-Riyami AA Afifi MM (2003). Prevalence and correlates of obesity and central obesity among Omani adults. Saudi Med J, 24: 641–646. 12847595

[32] Abdul-Rahim H Holmboe-Ottesen G Stene L (2003). Obesity in a rural and an urban Palestinian West Bank population. Int J Obes Relat Metab Disord, 27: 140–146. 12532166 10.1038/sj.ijo.0802160

[33] Adediran OS Okpara IC Adeniyi OS (2012). Obesity prevalence and its associated factors in an urban and rural area of Abuja Nigeria. Global Advanced Research Journal of Medicine and Medical Sciences, 1: 237–241.

[34] Ghadiri-Anari A Jafarizadah M Zare A (2013). Prevalence of obesity and overweight among adults in Iranian population (Yazd Province). Iranian Journal of Diabetes and Obesity, 5: 67–70.

[35] Bell AC Ge K Popkin BM (2002). The road to obesity or the path to prevention: motorized transportation and obesity in China. Obes Res, 10: 277–283. 11943837 10.1038/oby.2002.38

[36] Misra A Singhal N Khurana L (2010). Obesity, the metabolic syndrome, and type 2 diabetes in developing countries: role of dietary fats and oils. J Am Coll Nutr, 29(3 Suppl):289S–301S. 20823489 10.1080/07315724.2010.10719844

[37] World Health Organization (2003). Diet, nutrition and the prevention of chronic diseases. World Health Organ Tech Rep Ser, 916: 1–149. 12768890

[38] Wang K Wang D Pan L (2015). Prevalence of Obesity and Related Factors among Bouyei and Han Peoples in Guizhou Province, Southwest China. PloS One, 10: e0129230. 26075708 10.1371/journal.pone.0129230 PMC4468129

[39] Aoyagi K Kusano Y Takamura N (2006). Obesity and cardiovascular risk factors among men and women aged 40 years and older in a rural area of Japan. J Physiol Anthropol, 25: 371–375. 17213689 10.2114/jpa2.25.371

[40] Rashidy-Pour A Malek M Eskandarian R (2009). Obesity in the Iranian population. Obes Rev, 10: 2–6. 19021868 10.1111/j.1467-789X.2008.00536.x

[41] Garawi F Devries K Thorogood N (2014). Global differences between women and men in the prevalence of obesity: is there an association with gender inequality? Eur J Clin Nutr, 68: 1101–1106. 24918120 10.1038/ejcn.2014.86

[42] Maddah M Nikooyeh B (2008). Urban and rural differences in pregnancy weight gain in Guilan, Northern Iran. Matern Child Health J, 12: 783–786. 17694425 10.1007/s10995-007-0273-5

[43] Heseker H Schmid A (2000). Epidemiology of obesity. Ther Umsch, 57: 478–481. [Article in German] 11026082 10.1024/0040-5930.57.8.478

[44] Martínez-Ros M Tormo M Navarro C (2001). Extremely high prevalence of overweight and obesity in Murcia, a Mediterranean region in south-east Spain. Int J Obes Relat Metab Disord, 25:1372–80. 11571602 10.1038/sj.ijo.0801684

[45] Maziak W Ward KD Rastam S (2005). Extent of exposure to environmental tobacco smoke (ETS) and its dose-response relation to respiratory health among adults. Respir Res, 6: 13. 15701169 10.1186/1465-9921-6-13 PMC549073

[46] Stene L Giacaman R Abdul-Rahim H (2001). Original communications-obesity and associated factors in a palestinian west bank village population. Eur J Clin Nutr, 55: 805–811. 11528498 10.1038/sj.ejcn.1601230

[47] WHO (2000). Obesity: preventing and managing the global epidemic: World Health Organization. 11234459

[48] Fouad M Rastam S Ward K (2006). Prevalence of obesity and its associated factors in Aleppo, Syria. Prev Control, 2: 85–94. 18040524 10.1016/j.precon.2006.09.001 PMC2094121

[49] Al-Nuaim AA Bamgboye EA Al-Rubeaan KA (1997). Overweight and obesity in Saudi Arabian adult population, role of sociodemographic variables. J Community Health, 22: 211–223. 9178120 10.1023/a:1025177108996

[50] Kac G Velásquez-Meléndez G Coelho MAS (2001). Factors associated with abdominal obesity among childbearing-age women. Rev Saude Publica, 35: 46–51. 11285517 10.1590/s0034-89102001000100007

[51] Musaiger A Al-Mannai M (2001). Weight, height, body mass index and prevalence of obesity among the adult population in Bahrain. Ann Hum Biol, 28: 346–350. 11393341 10.1080/030144601300119151

[52] Sibai AM Hwalla N Adra N (2003). Prevalence and covariates of obesity in Lebanon: findings from the first epidemiological study. Obes Res, 11: 1353–1361. 14627756 10.1038/oby.2003.183

[53] Lipowicz A Gronkiewicz S Malina RM (2002). Body mass index, overweight and obesity in married and never married men and women in Poland. Am J Hum Biol, 14: 468–475. 12112568 10.1002/ajhb.10062

[54] Ersoy C Imamoglu S (2006). Comparison of the obesity risk and related factors in employed and unemployed (housewife) premenopausal urban women. Diabetes Res Clin Pract, 72: 190–196. 16298012 10.1016/j.diabres.2005.10.010

[55] Dinsa G Goryakin Y Fumagalli E (2012). Obesity and socioeconomic status in developing countries: a systematic review. Obes Rev, 13: 1067–1079. 22764734 10.1111/j.1467-789X.2012.01017.x PMC3798095

[56] Vedana EHB Peres MA Neves Jd (2008). Prevalence of obesity and potential causal factors among adults in southern Brazil. Arq Bras Endocrinol Metabol, 52: 1156–1162. 19082304 10.1590/s0004-27302008000700012

[57] Ghassemi H Harrison G Mohammad K (2002). An accelerated nutrition transition in Iran. Public Health Nutr, 5: 149–155. 12027278 10.1079/PHN2001287

[58] Maddah M Eshraghian M Djazayery A (2003). Association of body mass index with educational level in Iranian men and women. Eur J Clin Nutr, 57: 819–823. 12821881 10.1038/sj.ejcn.1601615

[59] Shukla H Gupta P Mehta H (2002). Descriptive epidemiology of body mass index of an urban adult population in western India. J Epidemiol Community Health, 56: 876–880. 12388581 10.1136/jech.56.11.876 PMC1732045

[60] Lahti-Koski M Pietinen P Heliövaara M (2002). Associations of body mass index and obesity with physical activity, food choices, alcohol intake, and smoking in the 1982–1997 FINRISK Studies. Am J Clin Nutr, 75: 809–817. 11976153 10.1093/ajcn/75.5.809

[61] Farahani LA Asadi-Lari M Mohammadi E (2015). Community-based physical activity interventions among women: a systematic review. BMJ Open, 5: e007210. 10.1136/bmjopen-2014-007210 PMC4390687 25833668

[62] Schoenborn CA Adams PF Barnes PM (2002). Body weight status of adults: United States, 1997–98. Adv Data, (330):1–15. 12661975

[63] Rissanen A Heliövaara M Knekt P (1991). Determinants of weight gain and overweight in adult Finns. Eur J Clin Nutr, 45: 419–430. 1959514

[64] Abete I Astrup A Martínez JA (2010). Obesity and the metabolic syndrome: role of different dietary macronutrient distribution patterns and specific nutritional components on weight loss and maintenance. Nutr Rev, 68: 214–231. 20416018 10.1111/j.1753-4887.2010.00280.x

[65] Halton TL Hu FB (2004). The effects of high protein diets on thermogenesis, satiety and weight loss: a critical review. J Am Coll Nutr, 23: 373–385. 15466943 10.1080/07315724.2004.10719381

[66] Guttmacher S Kelly PJ Ruiz-Janecko Y (2010). Community-based health interventions: John Wiley & Sons.

[67] Austin GL Ogden LG Hill JO (2011). Trends in carbohydrate, fat, and protein intakes and association with energy intake in normal-weight, overweight, and obese individuals: 1971–2006. Am J Clin Nutr, 93: 836–843. 21310830 10.3945/ajcn.110.000141

